# Utero-Placental Immune Milieu during Normal and Aglepristone-Induced Parturition in the Dog

**DOI:** 10.3390/ani11123598

**Published:** 2021-12-19

**Authors:** Miguel Tavares Pereira, Renata Nowaczyk, Selim Aslan, Serhan S. Ay, Mariusz P. Kowalewski

**Affiliations:** 1 Institute of Veterinary Anatomy, Vetsuisse Faculty, University of Zurich (UZH), 8057 Zurich, Switzerland; kowalewski@vetanat.uzh.ch; 2 Department of Biostructure and Animal Physiology, Division of Histology and Embryology, Faculty of Veterinary Medicine, Wroclaw University of Environmental and Life Sciences, 50-366 Wroclaw, Poland; renata.nowaczyk@upwr.edu.pl; 3 Department of Obstetrics and Gynaecology, Faculty of Veterinary Medicine, Near East University, Nicosia 99138, North Cyprus, Turkey; selim.aslan@neu.edu.tr; 4 Department of Obstetrics and Gynecology, Ondokuz Mayıs University, Atakum 55200, Samsun, Turkey; serhan.ay@gmail.com; 5 Center for Clinical Studies (ZKS), Vetsuisse Faculty, University of Zurich (UZH), 8057 Zurich, Switzerland

**Keywords:** dog (*Canis lupus familiaris*), immune system, utero-placental, parturition, aglepristone

## Abstract

**Simple Summary:**

The tolerance of the maternal immune system towards the embryo is essential for the success of pregnancy in all mammals. The uterine immunological milieu is modulated in a species-dependent manner, and pro-inflammatory responses are observed in the uterus during parturition in several species. An analogous situation was suggested for the dog. Nevertheless, details regarding immune signaling in the canine utero-placental compartments remain veiled. The present investigation of gene expression and immunolocalization of several immune-related factors revealed moderate utero-placental activity during mid-pregnancy (maintenance period). However, several immune factors were upregulated during parturition, suggesting an increased incidence of cells involved in tissue remodeling and/or immune regulation. The involvement of progesterone in these mechanisms was further assessed by using samples from mid-pregnant dogs treated with the progesterone receptor blocker, aglepristone. Similarities were observed in the expression pattern of several immune factors between natural and induced parturition, supporting the involvement of progesterone signaling in the modulation of the uterine immune milieu. This study provides the basis for further investigations regarding the immune regulation of parturition in the dog. Furthermore, differences observed between natural and induced parturition could be related to different placental maturation and/or functional characteristics of aglepristone, and might be of clinical relevance.

**Abstract:**

Maternal immunotolerance is required for the maintenance of pregnancy, in sharp contrast with the uterine pro-inflammatory activity observed during parturition in several species. Correspondingly, in the dog, increased immune signaling at term has been suggested, but a deeper understanding of the uterine immune milieu is still missing. Thus, the availability of 30 immune-related factors was assessed in utero-placental samples collected during post-implantation (days 18–25 of pregnancy) and mid-gestation (days 35–40) stages, and at the time of prepartum luteolysis. Gene expression and/or protein localization studies were employed. Samples collected from antigestagen (aglepristone)-treated dogs were further analyzed. Progression of pregnancy was associated with the downregulation of *IL1β* and upregulation of *IL10* (*p* < 0.05) at mid-gestation. When compared with mid-gestation, a higher availability of several factors was observed at term (e.g., *CD206*, *CD4*, *TLR4*). However, in contrast with natural parturition, *MHCII*, *CD25*, *CCR7*, *TNFα*, *IDO1* and *AIF1* were upregulated after aglepristone treatment (*p* < 0.05), but not *TNFR1* or *CCL13* (*p* > 0.05). Altogether, these results show an increased immune activity during canine parturition, involving, i.a., M2 macrophages, Treg and Th cells, with strong support for progesterone-mediated immunomodulation. Furthermore, differences between term and induced parturition/abortion could relate to differences in placental maturation towards parturition and/or functional traits of antigestagens.

## 1. Introduction

A successful pregnancy depends on the balanced immune response of the uterus towards the semiallogeneic conceptus while remaining sensitive to allogenic pathogens. Indeed, a disproportionate immune response, resulting in the presence of inflammatory signals at the feto-maternal interface during the maintenance of pregnancy, is frequently associated with gestational complications, e.g., preterm parturition or preeclampsia in humans [1,2]. On the other hand, the inflammatory reaction still plays an important role in reproductive events, including ovulation, implantation and also parturition [3,4].

The concept of the maintenance of pregnancy as an anti-inflammatory uterine state, in contrast with the pro-inflammatory event of parturition, is further supported by the immunosuppressive roles of progesterone (P4) [5]. In fact, the periparturient stage is frequently associated with increased systemic inflammatory signals, e.g., in humans, mares and sows [6,7,8]. Importantly, whereas several endocrine mechanisms are species-specific, locally, i.e., intrauterine, decreased P4 signaling is a hallmark of parturition in most mammals. This can be associated with a functional P4 withdrawal in the placenta, as observed in humans (reviewed in [9]), or its decreased production following PGF2α-induced luteolysis in species such as the pig or cow [10,11]. In the dog, trophoblast-derived PGF2α is involved in the parturition cascade due to its luteolytic and presumably myocontractile activities [12,13,14,15,16]. The absence of steroidogenic activity in the placenta is characteristic of dogs and establishes the corpus luteum as the sole source of P4 needed for the maintenance of pregnancy [17,18]. Accordingly, PGF2α-induced luteolysis leads to an abrupt decrease in circulating sex steroids, P4 and estradiol-17β (E2), with parturition taking place 12–48 h later [16,18,19]. The role of the inhibition of P4 signaling in the canine parturition cascade is further highlighted by the effects observed upon functional suppression of the P4 nuclear receptor (PGR) with antigestagens (e.g., aglepristone), unequivocally resulting in preterm termination of pregnancy/abortion, associated with increased PGF2α release (reviewed in [20]).

With regard to local immune events during parturition, the ripening of the cervix and increased contractility of the myometrium are associated with pro-inflammatory signals and the infiltration of immune cells [21,22,23,24,25]. As for the placenta, most knowledge regarding local changes in the uterine immune milieu during parturition derives from research on humans. Accordingly, in human decidua, labor is frequently associated with increased expression of pro-inflammatory interleukins, i.a., IL1β, IL6, IL8 and TNFα [24,26,27]. Furthermore, several studies reported an increased presence of macrophages and lymphocytes during parturition in humans [28,29,30,31]. In this regard, whereas macrophages with immunomodulatory and/or tissue remodeling activities (M2) are the predominant phenotype during the maintenance of pregnancy in humans, an increased presence of pro-inflammatory M1 macrophages (involved in Th1 response) appeared to be required for the initiation of parturition [30]. Similarly, in cattle, an increased utero-placental infiltrate of immune cells, involving M1 macrophages, neutrophils and T lymphocytes, is observed at the time of parturition [32,33,34]. Furthermore, an increased number of lymphocytes has been described in later stages of pregnancy in the interplacentomal epithelium of cows, ewes and deer [35]. This increased presence of immune cells in the embryo–maternal interface appears to play an important role in the detachment of fetal membranes, as shown in cattle [36,37].

Despite the great importance of the dog as a companion animal, there is not much information available about this species’ utero-placental immune system. Recently, the dynamic changes in the uterine immune milieu of the dog during the peri-implantation period have been reported [38]. Whereas the presence of the embryo before implantation was associated with moderate pro-inflammatory activity, implantation was associated with the presence of a local anti-inflammatory signal, possibly involving the activity of regulatory T and NK cells [38]. Also the early placentation period appeared to be associated with decreased immune activity [38]. With regard to parturition, a recent transcriptional analysis from our group described an increased immune activity in the canine placenta during normal and antigestagen-induced parturition/abortion [39]. Although uterine involution in dogs appears to be associated with an endometrial immune infiltrate consisting mostly of T lymphocytes [40], further details about the uterine immune milieu during the maintenance and termination of canine pregnancy are still missing. Therefore, we investigated the expression and/or localization of several immune factors in canine utero-placental samples from mid- and late gestation. Possible P4-mediated effects were addressed by using samples from mid-gestation bitches in which abortion was induced with the PGR blocker aglepristone.

## 2. Materials and Methods

### 2.1. Tissue Samples Collection and Preservation

Samples from 23 crossbreed and clinically healthy bitches aged 2–8 years old were used, all derived from previous projects [39,41,42]. The presence of spontaneous ovulation was determined when circulating P4 concentrations were above 5 ng/mL, detected with radioimmunoassay. Mating (day 0 of pregnancy) occurred 2–3 days after ovulation and samples of utero-placental compartments were collected by routine ovariohysterectomy at different stages of pregnancy. Considering that implantation occurs around 17 days after mating in the dog [19,43,44], post-implantation (Post-Imp, *n* = 5) samples were collected between days 18–25 of pregnancy, while samples from the matured placenta, i.e., mid-gestation (Mid-Gest, *n* = 5), were collected on days 35–40 of pregnancy. Samples from prepartum luteolysis (Lut, *n* = 3) were collected during the active prepartum P4 decline. To determine this stage, pregnancies were monitored endocrinologically and clinically, and utero-placental samples were collected when P4 circulating amounts dropped below 3 ng/mL. Additionally, abortion was induced in 10 animals during mid-gestation (days 40–45) with aglepristone (Alizine, Virbac, Bad Oldesloe, Germany), a type II blocker of the P4 nuclear receptor. To achieve abortive effects, 10 mg/kg of body weight was administered subcutaneously twice with a 24 h interval between doses, following the manufacturer’s protocol. Ovariohysterectomy was then performed 24 h (24 h Agle) or 72 h (72 h Agle) after the second administration of aglepristone (*n* = 5/group).

Immediately after surgery, samples were washed with PBS, trimmed from surrounding connective tissue, immersed in RNAlater (Ambion Biotechnology GmbH, Wiesbaden, Germany) for 24 h at 4 °C and then stored at −80 °C until total RNA isolation. For histological analysis, samples were instead fixed in 10% neutral phosphate-buffered formalin for 24 h and then processed and paraffin-embedded following routine procedures.

### 2.2. Total RNA Isolation, High Capacity Reverse Transcription, Pre-Amplification of cDNA and Semi-Quantitative Real-Time TaqMan PCR

TRIzol reagent (Invitrogen, Carlsbad, CA, USA) was used to isolate total RNA, following the manufacturer’s instructions. The quantity and purity of RNA were assessed with a NanoDrop 2000 spectrophotometer (ThermoFisher Scientific AG, Reinach, Switzerland), and possible contaminating genomic DNA was degraded using the RQ1 RNA-free DNase kit (Promega, Dübendorf, Switzerland). Reverse transcription and pre-amplification of cDNA were performed following the protocol supplied by the manufacturer of the High Capacity cDNA Reverse Transcription Kit (Applied Biosystems by ThermoFisher Scientific, Foster City, CA, USA) and as previously described [45], using 10 ng of total RNA per sample. The cDNA was enriched for the selected targets by mixing with the TaqMan PreAmp Master Mix Kit (Applied Biosystems) and pooled predesigned commercially available TaqMan systems (obtained from Applied Biosystems) and self-designed TaqMan systems ordered from Microsynth AG (Balgach, Switzerland) composed of primers and 6-carboxyfluorescein (6-FAM) and 6-carboxytetramethylrhodamine (TAMRA) probes. The full list of TaqMan systems and self-designed primers and 6-FAM and TAMRA probes is presented in Table S1. Gene expression of all 29 evaluated factors was assessed by semi-quantitative real-time TaqMan PCR, as previously described [45,46,47]. The reactions were run in an automated ABI PRISM 7500 Sequence Detection System (Applied Biosystems) with FastStart Universal Probe Master (ROX, Roche Diagnostics AG, Rotkreuz, Switzerland). All reactions were performed in duplicate, with TaqMan systems presenting an efficiency of reaction close to 100%. Not reverse-transcribed RNA or water were used instead of cDNA as negative controls. Obtained values were calibrated with the average expression of all samples and relative gene expression was then quantified using the ΔΔCt method. For normalization of gene expression, 3 reference genes were initially evaluated in all samples: *GAPDH, β-ACTIN* and *CYCLOPHILIN*. *β-ACTIN* and *GAPDH* were determined as being the more stable in all samples by the online tool RefFinder [48] and, thus, used as reference genes in the relative gene expression evaluation. Numerical values for gene expression are presented as mean +/− standard error of the mean (SEM). Statistical evaluation of these results was performed with the one-way analysis of variance (ANOVA) followed by Tukey–Kramer multiple comparisons post-test, using GraphPad 2.06 (GraphPad Software Inc., San Diego, CA, USA). Possible significant differences between CD4 and CD8 expression at each analyzed time-point were evaluated by applying the unpaired two-tailed Student’s *t*-test. Statistical significance was considered when *p* was lower than 0.05.

### 2.3. Immunohistochemical Staining

The immunohistochemical detection of selected proteins was performed with the standard indirect immunoperoxidase method, using our previously published protocol [47,49]. Sections with 2–3 μm thickness of formalin-fixed and paraffin-embedded tissue were deparaffinized with xylol and rehydrated with decreasing concentrations of ethanol. After a wash with tap water, slides were immersed in citrate buffer (pH = 6) and antigen retrieval with heat was performed in a microwave oven. Endogenous peroxidases were then quenched with 0.3% hydrogen peroxide diluted in methanol and nonspecific binding sites were blocked with 10% serum from goat or horse, depending on the secondary antibody used. Incubation with primary antibodies was performed overnight at 4 °C. All washing steps were performed with IHC buffer (0.8 mM Na_2_HPO_4_, 1.74 mM KH_2_PO_4_, 2.68 mM KCl, 137 mM NaCl; pH = 7.2) containing 0.3% Triton X. The full list of antibodies used, and dilutions, can be found in Table S2. A negative control was performed with isotype reactions, where primary antibodies were replaced with non-immune IgG from respective species and at a similar concentration (rabbit IgG I-1000, goat IgG I-5000 and mouse IgG I-2000, Vector Laboratories Inc., Burlingame, CA, USA). After incubation with secondary antibody (horse anti-goat IgG BA-9500, goat anti-rabbit IgG BA1000 or horse anti-mouse IgG BA-2000, Vector Laboratories Inc., Burlingame, CA, USA) and with streptavidin-peroxidase Vectastain ABC kit (Vector Laboratories Inc., Burlingame, CA, USA), antigen–antibody complexes were visualized with the liquid DAB + substrate kit (Dako Schweiz AG, Baar, Switzerland). Counterstaining was performed with hematoxylin. Slides were finally dehydrated in a series of increasing ethanol concentrations and mounted with Histokit (Assistant, Osterode, Germany). A qualitative assessment for the localization of positive signals and collection of representative pictures were performed using a Leica DMRXE light microscope equipped with a Leica DFC425 camera (Leica Microsystems, Wetzlar, Germany).

## 3. Results

### 3.1. Pregnancy and Term (Prepartum Luteolysis)

#### 3.1.1. Macrophages and Lymphocytes

**Figure 1:**
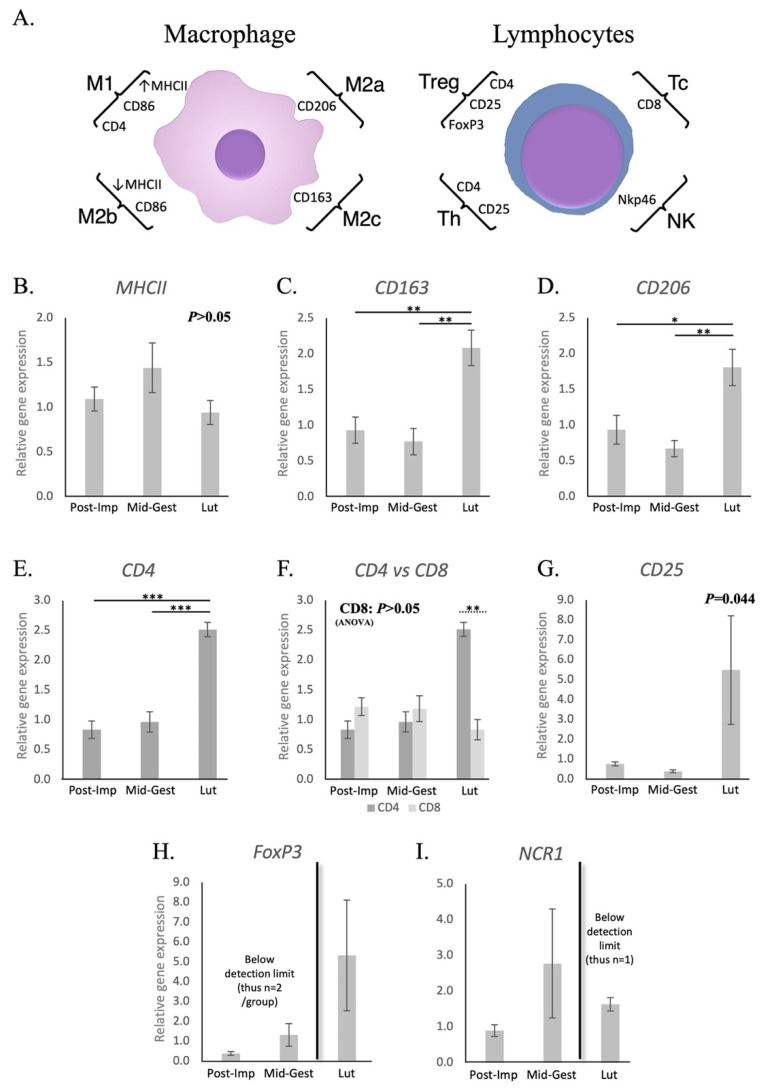
Schematic representation of surface markers selected for the characterization of subsets of macrophages and lymphocytes and their relative gene expression in the canine utero-placental compartment. (**A**) Surface markers evaluated to characterize the presence and/or localization of M1 (high MHCII expression, CD86 and CD4), M2a (CD206), M2b (low MHCII expression and CD86) and M2c (CD163) macrophages, and of T regulator (CD4, CD25 and FoxP3), T helper (CD4 and CD25), T cytotoxic (CD8) lymphocytes and natural killer cells (Nkp46, encoded by *NCR1*). (**B**–**I**) Relative gene expression as determined by semi-quantitative real-time (TaqMan) PCR ($\bar{X}$ +/− SEM). (**B**–**G**) To evaluate the effects of pregnancy progression, one-way ANOVA was applied, revealing: *p* = 0.298 for *MHCII*, *p* = 0.0038 for *CD163*, *p* = 0.0098 for *CD206*, *p* < 0.0001 for *CD4*, *p* = 0.411 for *CD8* and *p* = 0.044 for *CD25*. When *p* < 0.05, analysis was followed by a Tukey–Kramer multiple comparison post-test. (**F**) Comparison of relative gene expression between *CD4* and *CD8* at each stage of pregnancy was evaluated by applying Student’s unpaired two-tailed *t*-test. (**G**) No significant differences between groups were observed for *CD25* (*p* > 0.05). (**H**,**I**) Statistical analysis was not possible for *FoxP3* nor *NCR1* (expression frequently below detection limits in different groups). Bars with asterisks differ at: * *p* < 0.05, ** *p* < 0.01, *** *p* < 0.001. Post-Imp = post-implantation, Mid-Gest = mid-gestation, Lut = prepartum luteolysis.

As represented in Figure 1A, possible changes in the presence of different phenotypes of macrophages in the placenta were primarily investigated by assessing the transcriptional availability of the surface markers *MHCII*, *CD163* and *CD206*, whereas *CD4*, *CD8*, *NCR1*, *CD25* and *FoxP3* were investigated for lymphocytes. These markers were used in our previous publication [38], and were selected based on current literature [29,50,51,52,53,54,55,56]. No significant changes in the expression of *MHCII* could be observed in the utero-placental compartments during the progression and termination of pregnancy (*p* > 0.05, Figure 1B). However, transcript levels of *CD163* (*p* = 0.01, Figure 1C) and *CD206* (*p* < 0.05, Figure 1D) were the highest at the time of luteolysis, when compared with the post-implantation and mid-gestation stages. Likewise, the expression of *CD4* was higher at term than in earlier stages of pregnancy (*p* < 0.001, Figure 1E). In fact, transcriptional levels of *CD4* at prepartum luteolysis were significantly higher than those of *CD8* (*p* < 0.01, Figure 1F), the latter remaining stably expressed throughout all studied stages (*p* > 0.05, Figure 1F). Although stage-dependent effects were apparent for *CD25* (ANOVA *p* = 0.04), with an apparently increased expression at the time of luteolysis, no significant differences among the investigated stages could be identified by the post-hoc test (*p* > 0.05, Figure 1G). The transcriptional availability of *FoxP3* was frequently below the detection limits in most samples from the post-implantation and mid-gestation stages (Figure 1H), while this occurred for *NCR1* at the time of luteolysis (Figure 1I). This precluded any statistical analysis for both *FoxP3* and *NCR1*.

**Figure 2:**
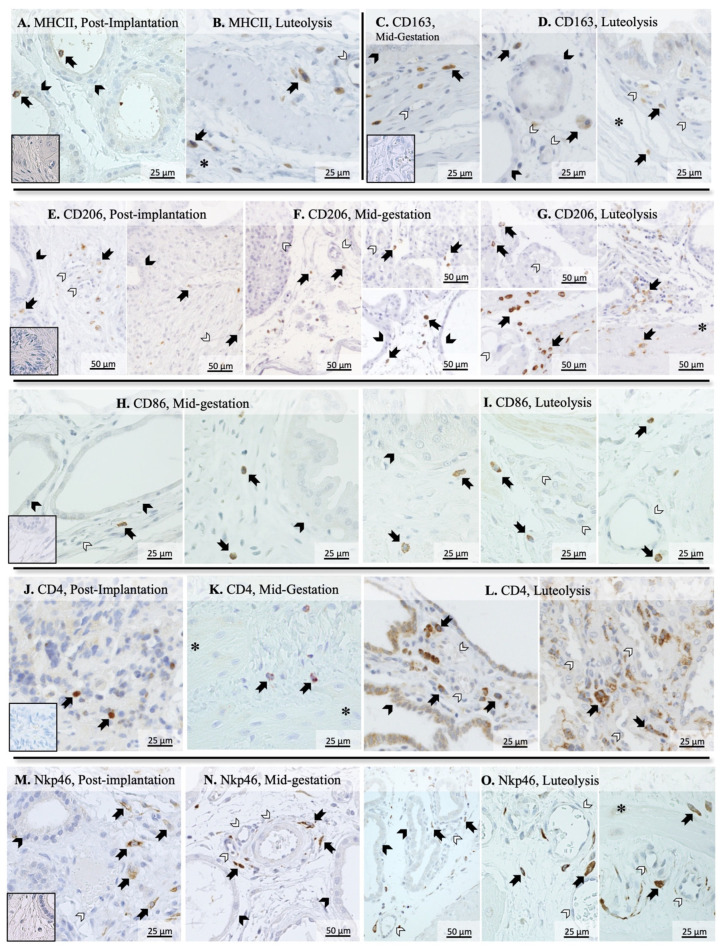
Immunohistochemical detection of selected surface markers of macrophages and lymphocytes in the canine placenta. MHCII-positive cells were identified within and around deep uterine glands during post-implantation (**A**) and in the myometrium during luteolysis (**B**). CD163 signals were observed in immune cells identified as macrophages located mostly in the supraglandular layer in mid-gestation (**C**), and around deep uterine glands and in the myometrium during luteolysis (**D**). During the post-implantation stage, CD206-positive macrophages were mainly localized in the supraglandular layer (**E**, left panel) and around deep uterine glands (**E**, right panel), with single cells also being present in the myometrium (**E**, right panel). During mid-gestation, these cells were localized not only around deep glands (**F**, right bottom panel), but also in the chorioallantoic membrane (**F**, left panel) and in the placental labyrinth (**F**, right top panel). An apparently increased number of cells staining for CD206 were localized in the chorioallantoic membrane (**G**, top left panel) and placental labyrinth (**G**, bottom left panel), with a lower number still being identified in deeper uterine layers (**G**, right panel). Macrophages stained against CD86 were localized in the connective tissue around deep uterine glands (**H**, left panel) and in the supraglandular layer (**H**, right panel) during mid-gestation. During luteolysis, these cells were located not only around uterine glands (**I**, left panel), but also in the chorioallantoic membrane around blood vessels (**I**, middle and right panels). Whereas CD4-positive cells were present in superficial layers of the endometrium during post-implantation (**J**), they were mostly identified in deeper layers of the endometrium and in the myometrium during later mid-gestation (**K**) and luteolysis (**L**, right panel). Furthermore, cells stained positively with CD4 were also localized in the chorioallantoic membrane during luteolysis (**L**, left panel). Finally, Nkp46-positive lymphocytes were mostly present in deeper layers of the endometrium during post-implantation (**M**) and mid-gestation (**N**). However, these cells could be localized in the placental labyrinth (**O**, left panel), around deep blood vessels in the endometrium (**O**, center panel) and in the myometrium (**O**, right panel) during luteolysis (solid arrow = macrophages; closed arrowhead = uterine gland; open arrowhead = blood vessel, asterisk = myometrium). No staining was observed in the isotype controls (inset in **A**,**C**,**E**,**G**,**J**,**M**).

Additional assessment of the utero-placental immune infiltrate during mid and late stages of pregnancy was performed using immunohistochemistry. MHCII-positive immune cells, identified as monocytes/macrophages, could be localized within uterine glands (Figure 2A) and in the connective tissue surrounding them during post-implantation (not shown). In addition, at the time of prepartum luteolysis, these cells were further localized in the myometrium (Figure 2B). Cells expressing CD163 were seldom in post-implantation samples (not shown), but an apparently increased presence was observed in later stages of pregnancy. Thus, during mid-gestation, CD163 positively stained macrophages were mainly detected in the supraglandular layer (Figure 2C), with single cells present in deeper uterine layers. In contrast, most positively stained cells were localized around deep uterine glands and within the myometrium during luteolysis (Figure 2D). CD206-positive cells appeared to be the dominant macrophage subpopulation in the uterus, being mainly distributed in the supraglandular layer and between deep uterine glands in post-implantation (Figure 2E), with single cells being also present in the myometrium. During mid-gestation, single CD206-positive macrophages could be observed within the chorioallantoic membrane (Figure 2F, left panel) and placental labyrinth (Figure 2F, top right panel), while numerous cells were localized in the supraglandular layer around uterine glands (Figure 2F, bottom right panel) and in the myometrium. The number of CD206-positive macrophages was apparently increased during luteolysis in the chorioallantoic membrane and placental labyrinth (Figure 2G, left panels), as well as around deep glands and in the myometrium (Figure 2G, right panel). Finally, macrophages expressing CD86 were localized within the connective tissue between deep uterine glands in mid-gestation (Figure 2H). However, during luteolysis, these cells were not only observed in the connective tissue surrounding uterine glands (Figure 2I, left panel), but also in the connective tissue around blood vessels in the chorioallantoic membrane (Figure 2I, middle and right panel).

The differentiation between macrophages and lymphocytes expressing CD4 was performed in our previous work [38], referring to the differential staining of consecutive slides and morphological differentiation. Regarding their immunolocalization, CD4-positive cells, mainly recognized as lymphocytes, were localized in superficial layers of the endometrium during post-implantation (Figure 2J), while single cells could be observed in deep layers of the endometrium and myometrium during mid-gestation (Figure 2K). During luteolysis, CD4-positive lymphocytes were detectable around deep uterine glands (Figure 2L, left panel) and in the placental labyrinth (Figure 2L, right panel). Furthermore, CD4-positive signals could also be observed in the glandular epithelium (Figure 2L, left panel). Finally, Nkp46 (encoded by *NCR1*) positively stained natural killer (NK) cells were localized in deep layers of the endometrium during post-implantation and mid-gestation (Figure 2M,N, respectively). However, a wider distribution could be observed at the time of luteolysis, with NK cells being observed in the placental labyrinth (Figure 2O, left panel), around blood vessels in the deep uterine layer (Figure 2O, center panel) and in the myometrium (Figure 2O, right panel).

#### 3.1.2. Cytokines and Other Immunomodulators

**Figure 3:**
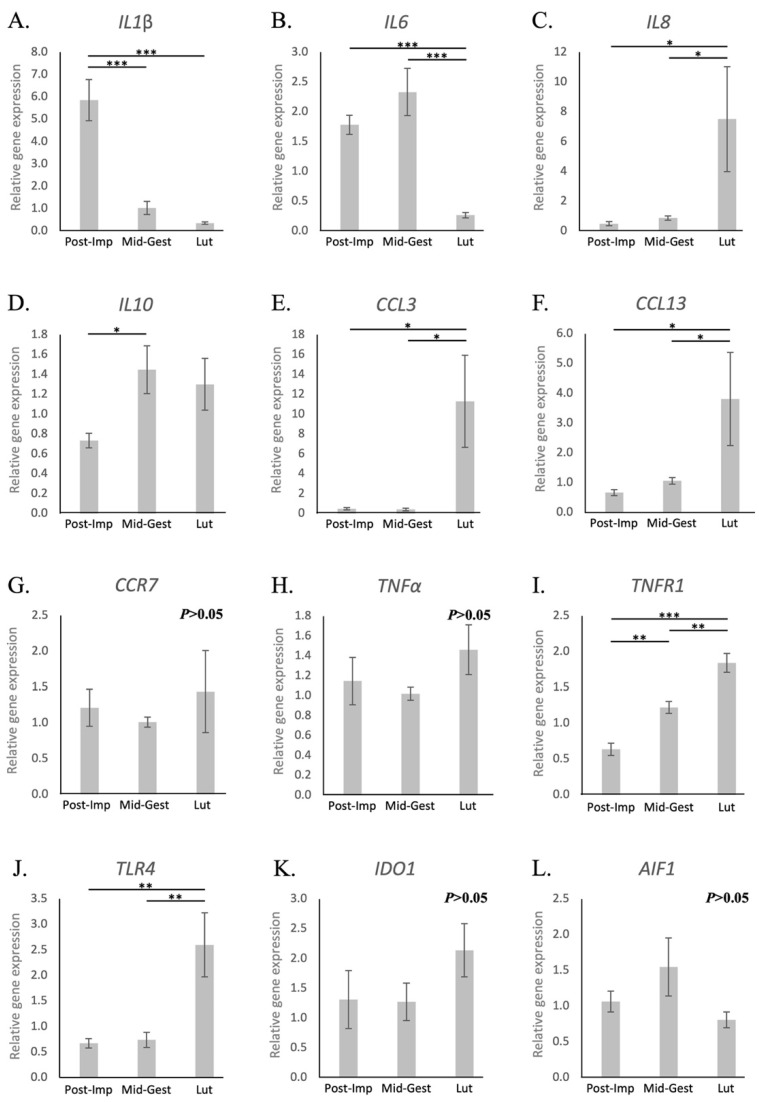
Relative gene expression of selected cytokines in the canine utero-placental compartment. (**A**–**L**) Relative gene expression as determined by semi-quantitative real-time (TaqMan) PCR ($\bar{X}$ +/− SEM). To evaluate the effects of pregnancy progression, one-way ANOVA was applied, revealing: *p* = 0.0002 for *IL1β*, *p* = 0.0017 for *IL6*, *p* = 0.0189 for *IL8*, *p* = 0.0469 for *IL10*, *p* = 0.0124 for *CCL3*, *p* = 0.0141 for *CCL13*, *p* = 0.666 for *CCR7*, *p* = 0.3615 for *TNFα*, *p* < 0.0001 for *TNFR1*, *p* = 0.0022 for *TLR4*, *p* = 0.39 for *IDO1* and *p* = 0.2692 for *AIF1*. When *p* < 0.05, analysis was followed by a Tukey–Kramer multiple comparison post-test. Bars with asterisks differ at: * *p* < 0.05, ** *p* < 0.01, *** *p* < 0.001. Post-Imp = post-implantation, Mid-Gest = mid-gestation, Lut = prepartum luteolysis.

Possible changes in the utero-placental immune milieu associated with the progression and termination of pregnancy were further evaluated by assessing the expression of different cytokines and other immune modulators, as well as of selected growth factors and markers of tissue remodeling. The availability of both *IL1β* and *-6* decreased with the passage of time. However, while samples collected at the post-implantation stage presented the highest expression of *IL1β* (*p* < 0.001, Figure 3A), *IL6* levels decreased only at the time of luteolysis (*p* < 0.001, Figure 3B). Conversely, the transcriptional availability of *IL8* was the highest at the time of luteolysis (*p* < 0.05, Figure 3C), whereas the expression of *IL10* increased from post-implantation to mid-gestation (*p* < 0.05), but remained unchanged during prepartum luteolysis (Figure 3D). In contrast with other interleukins, the expression of *IL12a* was frequently below detection limits in samples from the post-implantation and luteolysis periods (Figure S1A). As for chemokines, the availability of *CCL3* and *CCL13* was the highest at the time of luteolysis (*p* < 0.05, Figure 3E,F, respectively), whereas *CCR7* remained stably expressed at the investigated time points of pregnancy (*p* > 0.05, Figure 3G). With regard to the TNF system, *TNFα* remained stably expressed at the analyzed stages of pregnancy (*p* > 0.05, Figure 3H), contrasting with the progressive increase of *TNFR1* with the passage of time (*p* < 0.01, Figure 3I). Finally, the utero-placental transcriptional availability of *TNFR2*, as well as of *TGFβ*, remained stable throughout mid and late pregnancy (*p* > 0.05, Figure S1B,C, respectively). With regard to other factors involved in immune regulation, although *TLR4* expression was significantly increased at the time of luteolysis (*p* < 0.01, Figure 3J), no changes were observed for *IDO1* or *AIF1* (*p* > 0.05, Figure 3K,L, respectively).

Further, depending on the availability of antibodies, immunolocalization was performed for some selected factors. TNFα was expressed by cells identified as macrophages localized around deep uterine glands during mid-gestation (Figure S1D). Similarly, TNFR1 was expressed by macrophages present around deep uterine glands in mid-gestation and luteolysis (Figure S1E,F), in addition to their localization in the chorioallantoic membrane (Figure S1E, right panel). However, endothelial cells, trophoblast and epithelial glandular cells also expressed this receptor in both stages (Figure S1E, right panel, and S1F, left panel). TNFR2 was expressed by immune cells present in the chorioallantoic membrane and around deep uterine glands during mid-gestation and prepartum luteolysis (Figure S1G,H). IDO1-positive signals at mid-gestation were observed in macrophages distributed around deep uterine glands and in glandular epithelial cells and endothelium (Figure S1I). Interestingly, during luteolysis, IDO1-positive macrophages were observed in the chorioallantoic membrane and placental labyrinth (Figure S1J). AIF1 was expressed by cells identified as macrophages localized within the chorioallantoic membrane, and by endothelial and glandular epithelial cells at the mid-gestation stage (Figure S1K). While a similar location of positive signals could be observed at luteolysis, they were apparently weaker, and positively stained macrophages were further localized in the placental labyrinth (Figure S1L).

Finally, no significant time-dependent effects could be observed in the expression of the different tissue remodeling-related factors investigated, i.e., *IGF1*, *IGF2*, *ENG*, *CDH1*, *ECM2* and *MMP2* (*p* > 0.05, Figure S2). Nevertheless, a higher availability of these factors was apparent during mid-gestation and, in the case of *ECM2* and *MMP2*, also at the time of luteolysis.

### 3.2. Aglepristone-Induced Luteolysis

**Figure 4:**
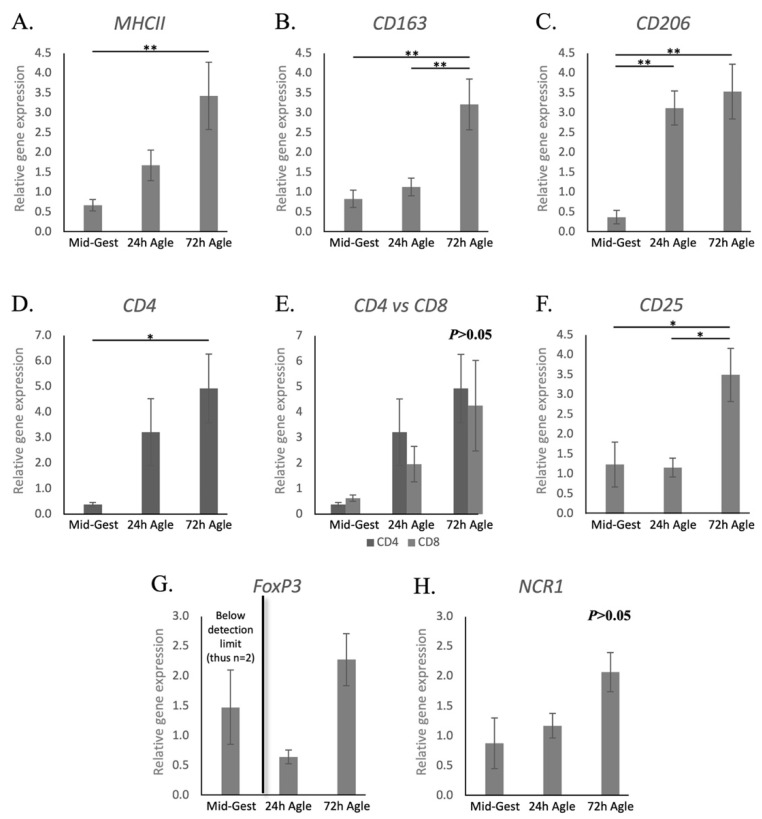
Relative gene expression of macrophages and lymphocytes surface markers in the canine utero-placental compartment during mid-gestation and aglepristone-induced luteolysis. (**A**–**H**) Relative gene expression as determined by semi-quantitative real-time (TaqMan) PCR ($\bar{X}$ +/− SEM). To evaluate the effects of the induction of luteolysis in mid-gestation dogs with aglepristone 24 and 72 h after treatment, one-way ANOVA was applied, revealing: *p* = 0.0078 for *MHCII*, *p* = 0.0021 for *CD163*, *p* = 0.0006 for *CD206*, *p* = 0.0329 for *CD4*, *p* = 0.071 for *CD8*, *p* = 0.0144 for *CD25* and *p* = 0.0566 for *NCR1*. When *p* < 0.05, analysis was followed by a Tukey–Kramer multiple comparison post-test. (**E**) Comparison of relative gene expression between *CD4* and *CD8* at each group was evaluated by applying Student’s unpaired two-tailed *t*-test. (**G**) Statistical analysis was not possible for *FoxP3* (expression during Mid-Gest frequently below detection limits). Bars with asterisks differ at: * *p* < 0.05, ** *p* < 0.01. Mid-Gest = mid-gestation, 24 h Agle = samples collected 24 h after the induction of luteolysis with aglepristone, 72 h Agle = samples collected 72 h after the induction of luteolysis with aglepristone.

Contrasting with natural parturition, the transcriptional availability of *MHCII* was increased 72 h after aglepristone treatment (*p* < 0.01, Figure 4A). On the other hand, as observed during natural parturition, aglepristone-induced abortion was associated with increased expression of the macrophage markers *CD163* after 72 h (*p* < 0.01, Figure 4B) and *CD206* 24 h after treatment (*p* < 0.01, Figure 4C). As for lymphocyte-related factors, *CD4* expression was upregulated 72 h after the second administration of aglepristone (*p* < 0.05, Figure 4D). Interestingly, transcriptional levels of *CD8* were apparently, but not significantly, increased by aglepristone (*p* > 0.05, Figure 4E). Furthermore, no significant differences between *CD4* and *CD8* transcriptional levels could be observed after the induction of abortion (*p* > 0.05, Figure 4E). The transcriptional availability of *CD25* was significantly higher 72 h after aglepristone treatment than at the other investigated stages (*p* < 0.05, Figure 4F). As observed during prepartum luteolysis, transcripts of *FoxP3* were detected after aglepristone treatment, but its irregular expression during mid-gestation precluded statistical analysis (Figure 4G). Finally, despite no significant differences, *NCR1* levels could be detected after aglepristone-induced luteolysis (Figure 4H), contrasting with its low expression at term.

**Figure 5:**
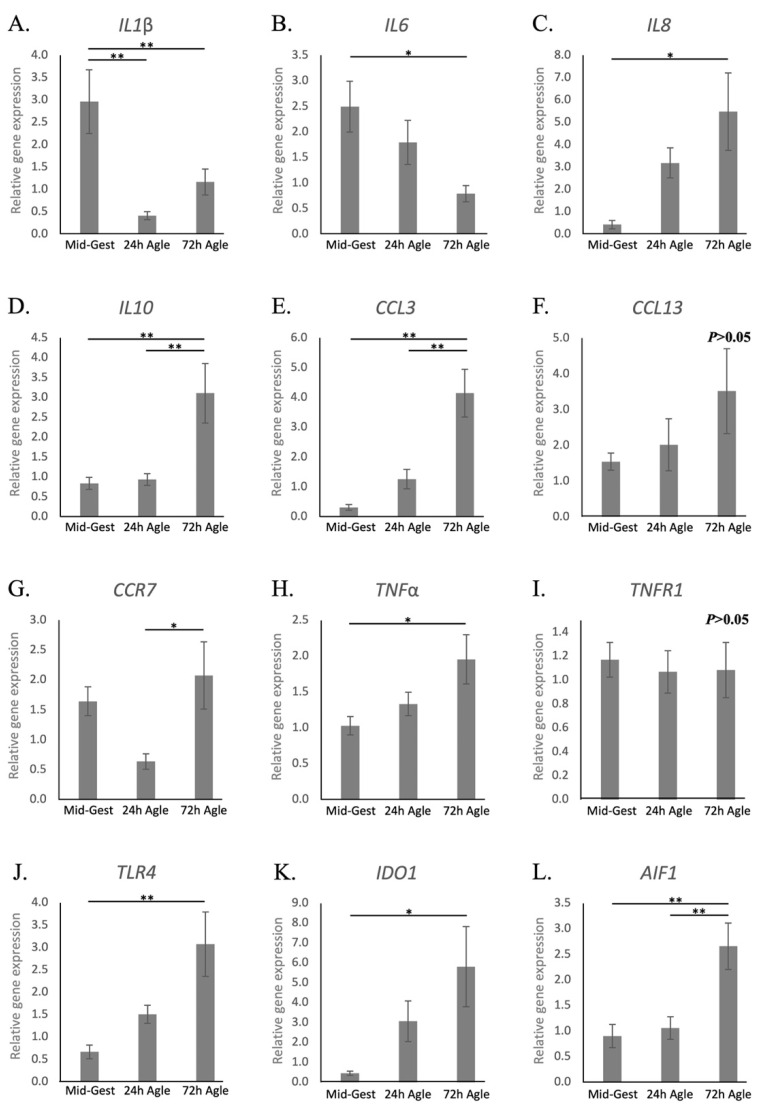
Relative gene expression of cytokines in the canine utero-placental compartments during mid-gestation and aglepristone-induced luteolysis. (**A**–**L**) Relative gene expression as determined by semi-quantitative real-time (TaqMan) PCR ($\bar{X}$ +/− SEM). To evaluate the effects of the induction of luteolysis in mid-gestation dogs with aglepristone 24 and 72 h after treatment, one-way ANOVA was applied, revealing: *p* = 0.0029 for *IL1β*, *p* = 0.0271 for *IL6*, *p* = 0.0204 for *IL8*, *p* = 0.0032 for *IL10*, *p* = 0.0011 for *CCL3*, *p* = 0.2643 for *CCL13*, *p* = 0.047 for *CCR7*, *p* = 0.0362 for *TNFα*, *p* = 0.9196 for *TNFR1, p* = 0.0111 for *TLR4*, *p* = 0.0281 for *IDO1* and *P* = 0.0033 for *AIF1*. When *P* < 0.05, analysis was followed by a Tukey–Kramer multiple comparison post-test. Bars with asterisks differ at: * *p* < 0.05, ** *p* < 0.01. Mid-Gest = mid-gestation, 24 h Agle = samples collected 24 h after the induction of luteolysis with aglepristone, 72 h Agle = samples collected 72 h after the induction of luteolysis with aglepristone.

Regarding interleukins, similarly to natural parturition, both *IL1β* and *-6* were downregulated during the termination of pregnancy by aglepristone (*p* < 0.05, Figure 5A,B, respectively), while the availability of *IL8* and *IL10* increased (*p* < 0.05, Figure 5C,D, respectively). The exception was *IL12a*, that was stably expressed after aglepristone-induced luteolysis (*p* > 0.05, Figure S3A), although being frequently below the detection limits at term. The chemokine *CCL3* was upregulated by the induced termination of pregnancy (*p* < 0.01, Figure 5E), but *CCL13* remained stably expressed (*p* > 0.05, Figure 5F). Furthermore, *CCR7* availability increased significantly between the 24 and 72 h groups (*p* < 0.05, Figure 5G), but not with regard to mid-gestation. Contrasting with samples collected at term, the transcriptional availability of *TNFα* increased 72 h after treatment (*p* < 0.05, Figure 5H), whereas *TNFR1* (Figure 5I), in addition to *TNFR2* and *TGFβ* (Figure S3B,C, respectively), remained unchanged (*p* > 0.05). With respect to other factors involved in immune regulation, not only *TLR4*, but also, in contrast with prepartum luteolysis, *IDO1* and *AIF1* were upregulated 72 h after treatment (*p* < 0.05, Figure 5J–L).

Finally, the expression of the growth factor *IGF1* was higher at 24 h but not at 72 h after treatment, when compared with mid-gestation (*p* < 0.01, Figure S3D), whereas *IGF2* remained stably expressed (*p* > 0.05, Figure S3E). As for the other factors involved in tissue remodeling, *ENG* increased in the 24 h after the administration of aglepristone, but not in samples from the 72 h group (*p* < 0.01, Figure S3F). Finally, the remaining evaluated factors, *CDH1*, *ECM2* and *MMP2*, remained unchanged during the aglepristone-induced termination of pregnancy, similar to natural parturition (*p* > 0.05, Figure S3G–I).

## 4. Discussion

Considering the sparse information available regarding the uterine immune milieu during later stages of gestation in the dog, the present work focused on the maintenance (mid-gestation) and termination (prepartum luteolysis) of pregnancy. Local pro-inflammatory activity in the uterus at the time of parturition appears to be a common phenomenon in eutherian mammals. This is also true for the dog [39]. Yet, differences in the uterine immune dynamics and signaling can be observed between different species. Our investigation of macrophage and T lymphocyte surface markers aimed to provide the first insights into possible changes in the cellular infiltrate associated with the progression and termination of canine pregnancy. Furthermore, the evaluation of cytokines and other immune modulators aimed to provide a more comprehensive characterization of the local immune milieu.

As recently shown [38], the post-implantation period presented the lowest transcriptional availability of several macrophage and lymphocyte surface markers, e.g., *MHCII*, *CD206*, *CD4*, *FoxP3* and *NCR1*, when compared with the earlier stages of pregnancy, represented by pre-implantation and implantation. This was proposed to be associated with a decreased activity of immune cells during the early placentation period, possibly involved in maternal tolerance mechanisms. Here, the progression of pregnancy towards mid-gestation, with a mature placenta, was marked by increased expression of the anti-inflammatory *IL10* and lower availability of the pro-inflammatory *IL1β*. This, together with the lack of modulatory stage-dependent effects observed for other factors, further supports the presence of decreased immune system activity during the maintenance of canine pregnancy, as previously suggested [38]. Accordingly, similar observations have been made in other mammals, e.g., human, rodents, horses, ruminants and opossum [34,57,58,59,60].

Prepartum luteolysis was characterized by increased transcriptional availability of *CD206* and *CD163* in utero-placental compartments, suggesting an increased utero-placental presence of macrophages with M2a (CD206-positive, involved in Th2 immunity) and M2c (CD163-positive, involved in tissue remodeling) features [29,50,61]. In contrast, *MHCII* and *IL10* were stably expressed during prepartum luteolysis, while *IL12a* transcripts were low. Macrophages with M1 characteristics, i.e., presenting Th1 immune activity, are associated with an increased production of IL12a and low levels of IL10, while an inverse situation is observed in M2 macrophages [29,61]. Thus, the expression patterns of *MHCII*, but also of *IL10* and -*12a*, appear to indicate a lower prevalence of M1 macrophages, when compared with M2a and/or M2c, in the uterine immune infiltrate at term. Furthermore, the localization pattern of immune cells also seemed to change from maintenance stages until prepartum. Different macrophage populations were mostly observed in the deep regions of the endometrium during maintenance stages. However, an increased presence of these cells in the superficial layers of the endometrium and/or within the placenta was apparent at term. In line with these observations in the dog, an increased infiltrate of macrophages in the feto-maternal interface has also been reported in humans, rats and cows [28,30,34]. However, the most prominent phenotypes of the invading macrophages in the dog appear to be mainly associated with tissue remodeling activities, contrasting with a higher prevalence of pro-inflammatory cells observed, e.g., in the cow [32,36], or humans, where cytokines related to M2 macrophages, such as IL10, are also downregulated [30,62].

Regarding lymphocytes, the increased transcriptional availability of *CD4* during luteolysis was accompanied by the apparently increased infiltration of CD4-positive cells in the canine placenta. The transcriptional availability of *CD4* at term was also significantly higher than that of *CD8*, which remained stably expressed throughout mid- and late pregnancy. This increased presence of CD4-positive T cells, but not of cytotoxic T lymphocytes (CD8+), in the canine placenta at term is in accordance with reports in humans [63]. Interestingly, CD4-positive signals were also observed in the glandular epithelium during mid-gestation. Although a similar localization of CD4 was previously observed in humans [64], suggesting a possible immunoactive role of glandular epithelial cells within the uterus, such a mechanism still needs further investigation. Regarding CD4+ lymphocyte subtypes, no significant changes in the utero-placental availability of *CD25*, expressed by Th and Treg lymphocytes [56], could be observed, although a tendency for its increase during luteolysis was apparent. Such a lack of effect could be the result of individual variations and/or the small sample size, since the significantly increased expression of *CD25* could be observed during aglepristone-induced parturition (discussed elsewhere). Nevertheless, a more complete characterization of CD4+ lymphocytes in the canine placenta, especially regarding different T helper subsets, is still required before further conclusions can be drawn. As for other CD4+ lymphocyte subtypes, *FoxP3*, a marker of regulatory T cells (Treg) [56], was expressed in all luteolysis samples, but was intermittently detected in earlier stages. Thus, despite the impossibility of statistical analysis, the *FoxP3* expression pattern appears to be related to an increased presence of Tregs in the utero-placental compartment, possibly involved in the modulation of inflammatory activity, during parturition. This could be further related to the lower amounts of the pro-inflammatory *IL6*, which, among other effects, can inhibit the differentiation of FoxP3+ Treg cells [33]. Finally, despite the lower incidence of NK cells in the utero-placental compartment at term, as implied by the decreased availability of *NCR1* (encoding for Nkp46), immunohistochemical analysis suggested their enrichment within the embryo–maternal interface. Still, the functional importance of these dynamics during the termination of parturition needs to be elucidated, as NK cells can be involved in a wide variety of activities, from cytotoxic activities to the modulation of immune response, or even vascularization, as widely described during implantation [52,57,65].

The suggested overall increased immune infiltrate in the utero-placental compartment during canine parturition is further supported by the increased availability of different chemoattractants, such as *IL8*, *CCL3* and *CCL13*. Moreover, there was higher availability of toll-like receptor 4 (*TLR4*), a factor supporting inflammatory activity [66], during prepartum luteolysis. Furthermore, despite the stable expression of *TNFα*, its local signaling appeared to be upregulated through the consecutive increased expression of its receptor 1 (*TNFR1*) from post-implantation until luteolysis. The role of TNFα in the canine placenta has still not been determined, but, in humans, it is associated with a plethora of functions, i.a., upregulation of IL8, induction of trophoblast apoptosis and increased production of matrix metalloproteinases (reviewed in [24]). In this way, TNFα might be important in the modulation of local immune activity and in the detachment of fetal membranes. Similar to TNFα, the pro-inflammatory IL1β is also involved, i.a., in the weakening of fetal membrane attachment in humans [67]. However, in contrast with observations from humans and other mammals [22,24,27,58], we detected a low level of expression of *IL1β* during prepartum luteolysis. As it is produced by pro-inflammatory macrophages, IL1β has been implicated in the initiation of parturition, e.g., by stimulating myometrial contractility through increased production of prostaglandins [63,68]. Although the decreased transcriptional availability of *IL1β* suggests that this might differ in the dog, it needs to be highlighted that the whole utero-placental compartment was used in the present study. Hence, the evaluation of immune signaling within the different uterine compartments, i.e., myometrium, endometrium, fetal–maternal interface and fetal membranes, is still required before any conclusions can be drawn in this regard.

Aglepristone competes with P4 in the binding to PGR, presenting a higher affinity to this receptor than P4 itself [69]. Thus, although aglepristone presents no direct effects on P4 levels, it presents high potential to disrupt PGR-mediated P4 signaling. The latter, i.e., the disruption of PGR-mediated effects, is expected to be potentiated by the function of type II antigestagens as transdominant repressors (reviewed in [20]). As an antigestagen, aglepristone is an effective abortifacient agent and the drug of choice in dogs (reviewed in [20]). Although most bitches have no to mild side effects, some animals experience uterine inflammatory conditions, such as endometritis [70,71]. Thus, the evaluation of the effects of aglepristone in the uterine immune milieu is of clinical relevance. Similar to what was observed during prepartum luteolysis, the expression of *CD163*, *CD206* and *CD4* was upregulated in the utero-placental samples after the induction of luteolysis with aglepristone. Furthermore, *CD25* was significantly increased, while *FoxP3* was detected after treatment, contrasting with mid-gestation. Regarding other immune factors, both *IL1β* and *IL6* transcriptional availability was decreased after the administration of aglepristone, whereas *TLR4*, *IL8* and *CCL3* were upregulated. Based on these observations, the termination of canine pregnancy, either at term or aglepristone-induced, appears to be characterized by similar immunoregulatory mechanisms, involving an increased presence of macrophages with M2a/c characteristics, as well as Treg and Th lymphocytes, in the utero-placental compartment. A relationship between these changes in the uterine immune milieu and decreased P4 signaling in the placenta, either due to prepartum or aglepristone-induced luteolysis, is also implied. Among other immunomodulatory effects, P4 signaling can modulate T cell activity through the increased expression of immunomodulatory transcription factors such as NFκB, or by inducing the production of the immunosuppressive progesterone-immunomodulatory blocking protein (PIBF1) (reviewed in [5,9]). However, the presence of such mechanisms still needs to be confirmed in the dog.

Despite several obvious similarities, the transcriptional availability of some factors diverged between the natural term and abortion. *MHCII*, *CCR7* and *IDO1*, all expressed by macrophages with the M1 phenotype [72], as well as *AIF1* and *TNFα*, were upregulated after the induction of abortion with aglepristone. Furthermore, no significant differences were observed between *CD4* and *CD8* expression, although *CD4* was significantly upregulated after aglepristone treatment. Finally, *NCR1* and *IL12a* were expressed in all aglepristone-treated samples, as also observed during mid-gestation, but were irregularly detected at term. Altogether, these results imply a higher presence of M1 macrophages, CD8+ lymphocytes and NK cells in the utero-placental compartment in preterm parturition samples than during prepartum luteolysis. The evaluation of such differences should take into consideration the different maturation status of the placentae (mid-gestation vs. term). Indeed, placentae collected at mid-gestation are exposed to the slowly decreasing P4 levels (due to slow luteal regression) for a shorter length of time and, in this way, are at a different preparation stage for parturition. Such maturation/time-dependent effects were previously discussed [39] and could further justify the transient increased expression of *IGF1* and *ENG* 24 h after aglepristone, but not at term, as compromising possible functional compensatory mechanisms. That said, the above-mentioned inhibitory mechanisms of the type II antigestagen aglepristone should also be considered. Similar to the ligand P4, type II antigestagens can also induce homodimerization of the PGR, leading to its binding in responsive elements of DNA. However, by inducing a different alteration of the PGR structure, they prevent the binding of coactivators and consequent activation of target gene expression (reviewed in [20]). Thus, by acting as an active PGR antagonist and transdominant repressor, aglepristone actively induces negative effects in PGR target genes. This contrasts with the passive decrease in PGR signaling due to the decreased availability of P4 at term and could further explain some of the differences we observed between natural and induced parturition.

## 5. Conclusions

**Figure 6:**
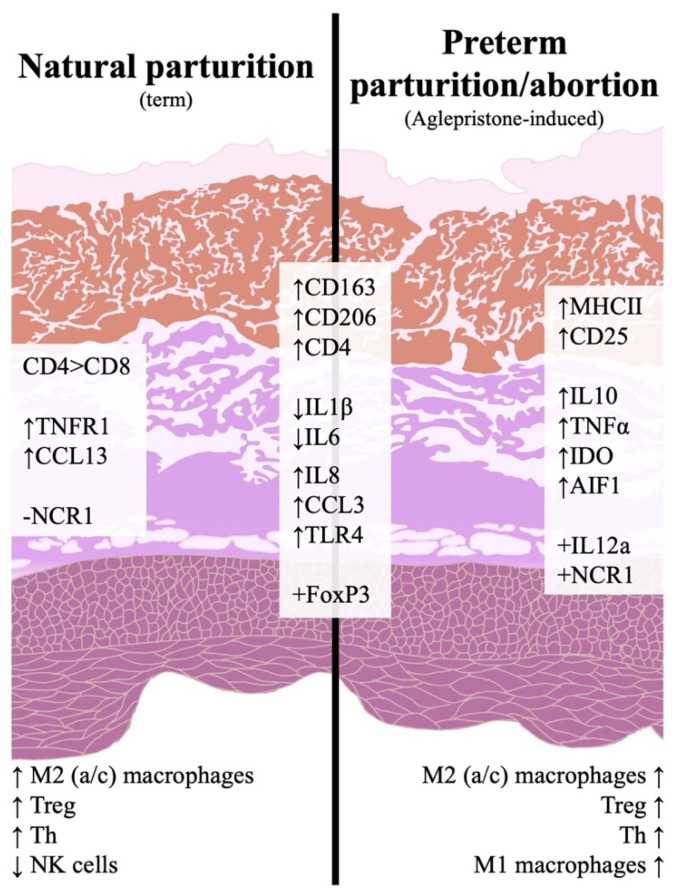
Schematic representation of the investigated immune events in the canine utero-placental compartment during natural or aglepristone-induced parturition. Arrows indicate increased (↑) or decreased (↓) transcriptional availability, and positive (+) and negative (−) indicate whether or not there was consistent transcriptional detection in parturition samples. Effects described at term are in comparison to post-implantation and/or mid-gestation, whereas abortion-related effects are in comparison to mid-gestation. Both natural and preterm parturition are associated with increased immune activity in the utero-placental compartment, involving the infiltration of macrophages with M2 characteristics (*CD206* for M2a and *CD163* for M2c), Treg cells (*Foxp3*) and Th cells (*CD4*, *CD25*). Term is also marked by an apparently lower presence of NK cells and a prevalence of CD4+ over cytotoxic CD8+ lymphocytes. In contrast, preterm parturition/abortion in response to aglepristone was associated with an increased presence of macrophages with M1 features (*MHCII*).

Overall, maintenance of pregnancy was associated with a low inflammatory activity in the utero-placental compartment. In contrast, an increased immune reaction was observed during natural and induced parturition/abortion, as summarized in Figure 6. This inflammatory activity appeared to comprise some species-specific mechanisms, i.a., the downregulation of *IL1β* and -6, and was mainly associated with an increased infiltration of macrophages involved in tissue remodeling and CD4+ lymphocytes, including Treg and Th. Aglepristone was further associated with an increased invasion of M1 macrophages and a higher incidence of NK cells than in samples collected at term. This might be related to the different maturation status of the placenta or to functional characteristics of antigestagens and acute withdrawal of P4/PGR signaling. Such differences represent important information for the clinical management of bitches when abortion is induced with antigestagens in mid-gestation. Finally, the present work provides some basis for further investigations into the involvement of the immune system in the parturition cascade in the dog.

## Data Availability

The data that support the findings of this study are available from the corresponding author upon reasonable request.

## Supplementary Materials

The following are available online at https://www.mdpi.com/article/10.3390/ani11123598/s1, Figure S1: Relative gene expression of IL12a, TNFR2 and TGFβ, and immunolocalization of TNF system members, IDO1 and AIF1, in the canine utero-placental compartment., Figure S2: Relative gene expression of factors involved in tissue remodeling in the canine utero-placental compartment, Figure S3: Relative gene expression of IL12a, TNFR2 and TGFβ and factors involved in tissue remodeling in the canine utero-placental compartment after aglepristone treatment, Table S1: List of gene symbols, corresponding gene names and TaqMan systems used for semi-quantitative real-time qPCR, Table S2: List of antibodies used in immunohistochemical staining.
