# Supplementary material for: Utero-Placental Immune Milieu during Normal and Aglepristone-Induced Parturition in the Dog
Source: Animals (Basel). 2021 Dec 19;11(12):3598. doi: 10.3390/ani11123598 (PMC8697996; doi:10.3390/ani11123598)
Supplement: Supplementary file 1 [file animals-11-03598-s001.zip › Sup figures/Table S1.pdf]

Table S1. List of gene symbols, corresponding gene names and TaqMan systems used for semi-quantitative real time qPCR

Gene	Name	Accession number	Primer sequence		Product length (bp)
MHC II	Major histocompatibility complex II	NM_001011723.1	Forward	5′-GGA GAG CCC AAC ATC CTC ATC-3′	90
			Reverse	5′-GGT GAC AGG GTT TCC ATT TCG-3′	
			TaqMan probe	5′-TCG ACA AGT TCT CCC CAC C-3′	
CD206/MCR1	Cluster of differentiation 206/mannose receptor C-Type 1	XM_005617091.3	Forward	5′-GGC AGG AAG ATT GTG TCG TCA T-3´	108
			Reverse	5´-TGG GCT GGG TTT GAG ATT TC-3´	
			TaqMan probe	5´-TGG GCA GAT CGA GCC TGC GAG-3´	
NCR1	Natural cytotoxicity triggering receptor 1	NM_001284448.1	Forward	5´-CTG GGA TCA CAC TGC CCA TAA T-3´	103
			Reverse	5´-CCT CTT CCT GCA AAG CCA GTA-3´	
			TaqMan probe	5´-CTT TCC TGG TCC TGA TGG CCC TCA-3´	
IL1β	Interleukin 1 beta	NM_001037971.1	Forward	5′-TGC CAA GAC CTG AAC CAC AGT-3′	97
			Reverse	5′-CTG ACA CGA AAT GCC TCA GAC T-3′	
			TaqMan probe	5′-CAT CCA GTT GCA AGT CTC CCA CCA GC-3′	
IL6	Interleukin 6	AF275796.1	Forward	5′-AAA GAG CAA GGT AAA GAA TCA GGA TG-3′	124
			Reverse	5′-GCA GGA TGA GGT GAA TTG TG-3′	
			TaqMan probe	5′-ACT CCT GAC CCA ACC ACA GAC GCC A-3′	
IL8/CXCL8	Interleukin 8/ C-X-C motif chemokine ligand 8	NM_001003200.1	Forward	5′-CCA CAC CTT TCC ATC CCA AA-3′	114
			Reverse	5′-CCA GGC ACA CCT CAT TTC CA-3′	
			TaqMan probe	5′-CTG AGA GTG ATT GAC AGT GGC CCA CAT TGT-3′	
TNFα	Tumor necrosis factor alpha	NM_001003244	Forward	5′-TGC CCT TCC ACC CAT GTG-3′	96
			Reverse	5′-AGG GCT CTT GAT GGC AGA GA-3′	
			TaqMan probe	5′-CCC ACA CCA TCA GCC GCT TCG-3′	
TNFR1	Tumor necrosis factor receptor 1	XM_849381	Forward	5′-TGT GTG GCT GCA GGA AGA AC-3′	114
			Reverse	5′-GCT TCT CTT GGC AGG AGA TCT-3′	
			TaqMan probe	5′-ACT CCA CCC TCT GCC TCA ATG GCA-3′	
TNFR2	Tumor necrosis factor receptor 2	XM_005617982	Forward	5′-CCA GCA GAG CGA GTA CTT CGA-3′	95
			Reverse	5′-TCG AGG TCT TGG TGC AGA AGA-3′	
			TaqMan probe	5′-CAT GTG TCC CCC TGG CTC CCA C-3′	
IDO-1	Indolamin 2,3-dioxygenase 1	XM_532793.5	Forward	5´-TGA TGG CCT TAG TGG ACA CAA G-3´	116
			Reverse	5´-TCT GTG GCA AGA CTT TTC GA-3´	
			TaqMan probe	5´-CAG CGC CTT GCA CGT CTG GC-3´	
AIF1	Allograft inflammatory factor 1	XM_532072.5	Forward	5´-CGA ATG CTG GAG AAA CTT GGT-3´	107
			Reverse	5´-TGA GAA AGT CAG AGT AGC TGAAGG T-3´	
			TaqMan probe	5´-TCC CCA AGA CCC ATC TGG AGC TCA A-3´	
GAPDH	Glyceraldehyde-3-phosphate dehydrogenase	AB028142.1	Forward	5′-GCT GCC AAA TAT GAC GAC ATC A-3′	75
			Reverse	5′-GTA GCC CAG GAT GCC TTT GAG-3′	
			TaqMan probe	5′-TCC CTC CGA TGC CTG CTT CAC TAC CTT-3′	
			TaqMan probe	5′-CAT GTG TCC CCC TGG CTC CCA C-3′	
CD163	Cluster of differentiation 163		Pre-designed assay from Applied Biosystems (Foster City, CA, USA) Prod.No. Cf02627321_m1		
CD4	Cluster of differentiation 4		Pre-designed assay from Applied Biosystems, Prod.No. Cf02627842_m1		
CD8	Cluster of differentiation 8		Pre-designed assay from Applied Biosystems, Prod.No. Cf02627888_m1		
CD25/IL2Rα	Cluster of differentiation 25/ interleukin 2 receptor alpha		Pre-designed assay from Applied Biosystems, Prod.No. Cf02623133_m1		
FoxP3	Forkhead Box P3		Pre-designed assay from Applied Biosystems, Prod.No. Cf02741703_m1		
IL10	Interleukin 10		Pre-designed assay from Applied Biosystems, Prod.No. Cf02624264_m1		
IL12α	Interleukin 12		Pre-designed assay from Applied Biosystems, Prod.No. Cf02628398_m1		
TGFβ	Transforming growth factor 1 beta		Pre-designed assay from Applied Biosystems, Prod.No. Cf02623324_m1		
CCL3	C-C motif chemokine ligand 3		Pre-designed assay from Applied Biosystems, Prod.No. Cf02671956_m1		
CCL13	C-C motif chemokine ligand 13		Pre-designed assay from Applied Biosystems, Prod.No. Cf02622470_mH		
CCR7	C-C motif chemokine receptor 7		Pre-designed assay from Applied Biosystems, Prod.No. Cf02654980_m1		
TLR4	Toll-like receptor 4		Pre-designed assay from Applied Biosystems, Prod.No. Cf02622203_g1		
IGF1	Insulin-like growth factor 1		Pre-designed assay from Applied Biosystems, Prod.No. Cf02627846_m1		
IGF2	Insulin-like growth factor 2		Pre-designed assay from Applied Biosystems, Prod.No. Cf02647136_m1		
ENG	Endoglin		Pre-designed assay from Applied Biosystems, Prod.No. Cf02658400_m1		
CDH1	Cadherin-1/epithelial cadherin (E-cadherin)		Pre-designed assay from Applied Biosystems, Prod.No. Cf02624268_m1		
ECM2	Extracellular matrix protein 2		Pre-designed assay from Applied Biosystems, Prod.No. Cf02641132_m1		
MMP2	Matrix metalloperoteinase 2		Pre-designed assay from Applied Biosystems, Prod.No. Cf02741675_m1		
β-actin	Beta-actin		Pre-designed assay from Applied Biosystems, Prod.No. Cf03023880_g1		
PPIA/ Cyclophilin	Peptidylprolyl isomerase A		Pre-designed assay from Applied Biosystems, Prod.No. Cf03986523_gH		
