# Supplementary material for: Utero-Placental Immune Milieu during Normal and Aglepristone-Induced Parturition in the Dog
Source: Animals (Basel). 2021 Dec 19;11(12):3598. doi: 10.3390/ani11123598 (PMC8697996; doi:10.3390/ani11123598)
Supplement: Supplementary file 1 [file animals-11-03598-s001.zip › Sup figures/Table S2.pdf]

Table S2. List of antibodies used in immunohistochemical staining

Target Protein	Product number	Antibody dilution
**MHCII**	ORB101661 (Biorbyt, Cambridge, UK)	1:200
**CD86**	ORB49101 (Biorbyt, Cambridge, UK)	1:400
**Nkp46**	ORB157934 (Biorbyt, Cambridge, UK)	1:400
**CD4**	AB125711 (Abcam, Cambridge, UK)	1:400
**TNFα**	AB6671 (Abcam, Cambridge, UK)	1:200
**TNFR1**	AB19139 (Abcam, Cambridge, UK)	1:200
**TNFR2**	AB15563 (Abcam, Cambridge, UK)	1:200
**IDO1**	LS-C174759 (LSBio, Seattle, WA, USA)	1:50
**AIF1**	LS-B2403 (LSBio, Seattle, WA, USA)	1:400
**CD206**	SC-376108 (Santa Cruz Biotecnology Inc., Santa Cruz, CA, USA)	1:400
**CD163**	DB045 (DB Biotech, Kosice, SK)	1:400
