# Supplementary figures and images for: Utero-Placental Immune Milieu during Normal and Aglepristone-Induced Parturition in the Dog
Source: Animals (Basel). 2021 Dec 19;11(12):3598. doi: 10.3390/ani11123598 (PMC8697996; doi:10.3390/ani11123598)

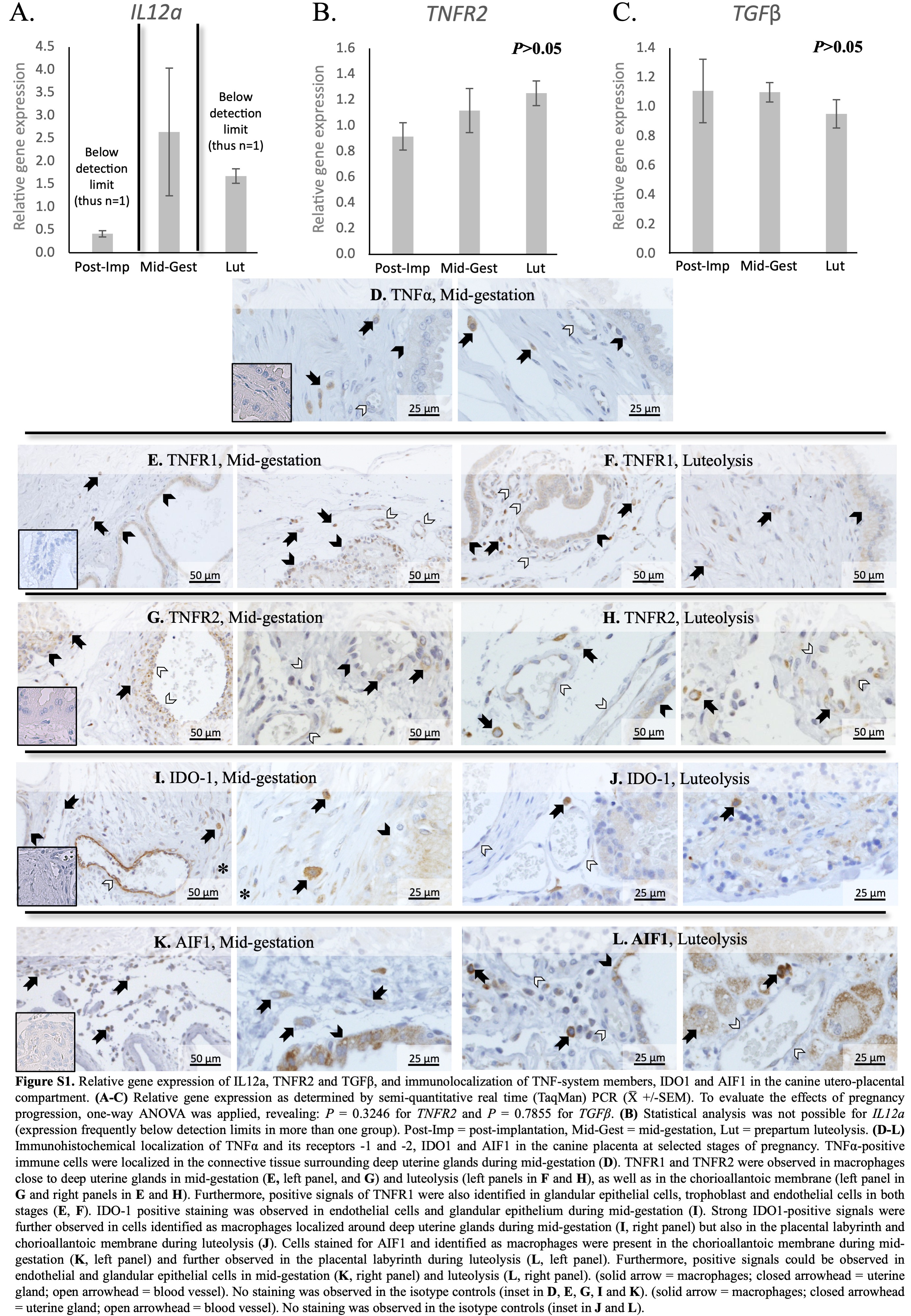

Supplement: Supplementary file 1 [file animals-11-03598-s001.zip › Sup figures/Figure S1.jpeg]

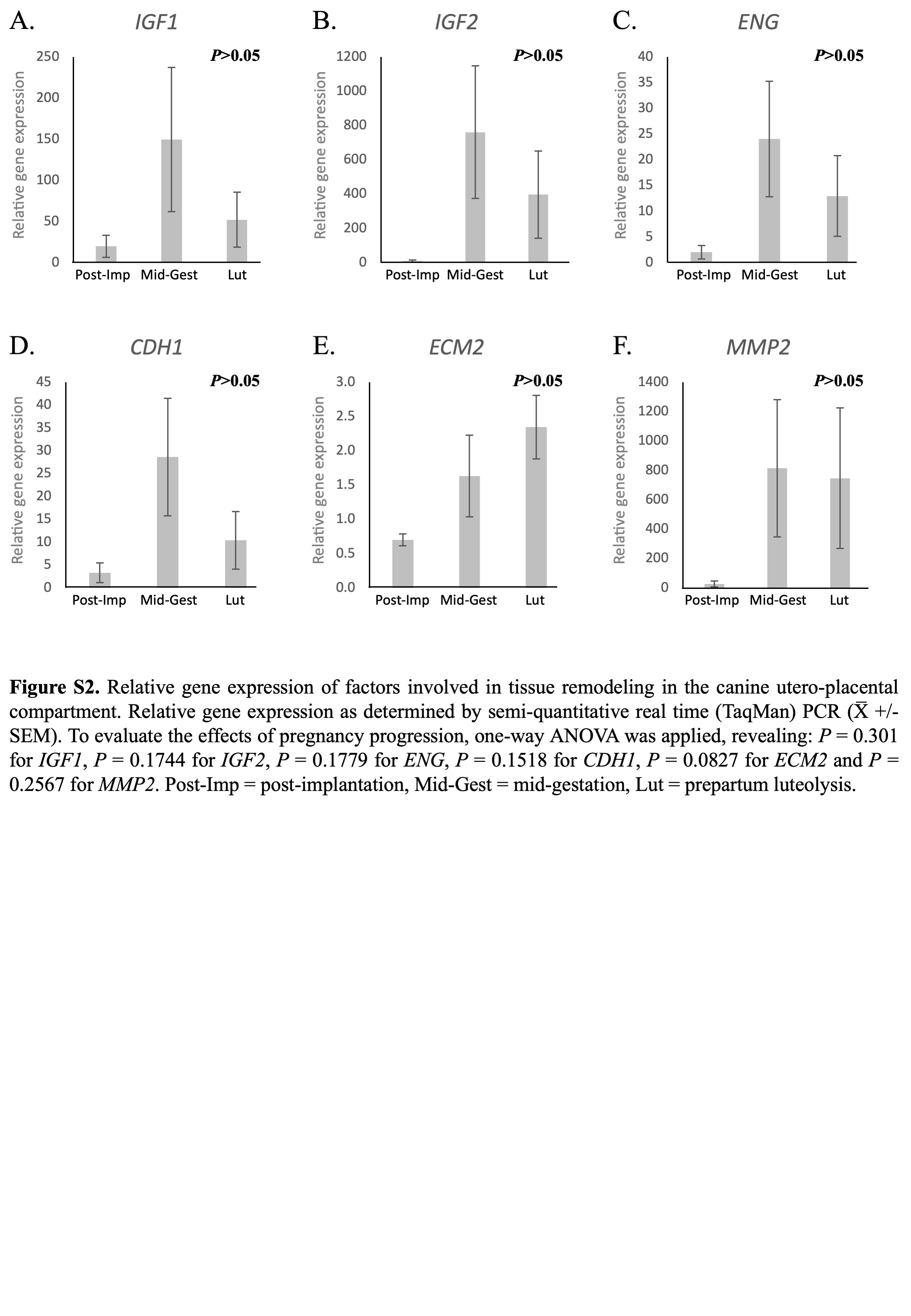

Supplement: Supplementary file 1 [file animals-11-03598-s001.zip › Sup figures/Figure S2.jpeg]

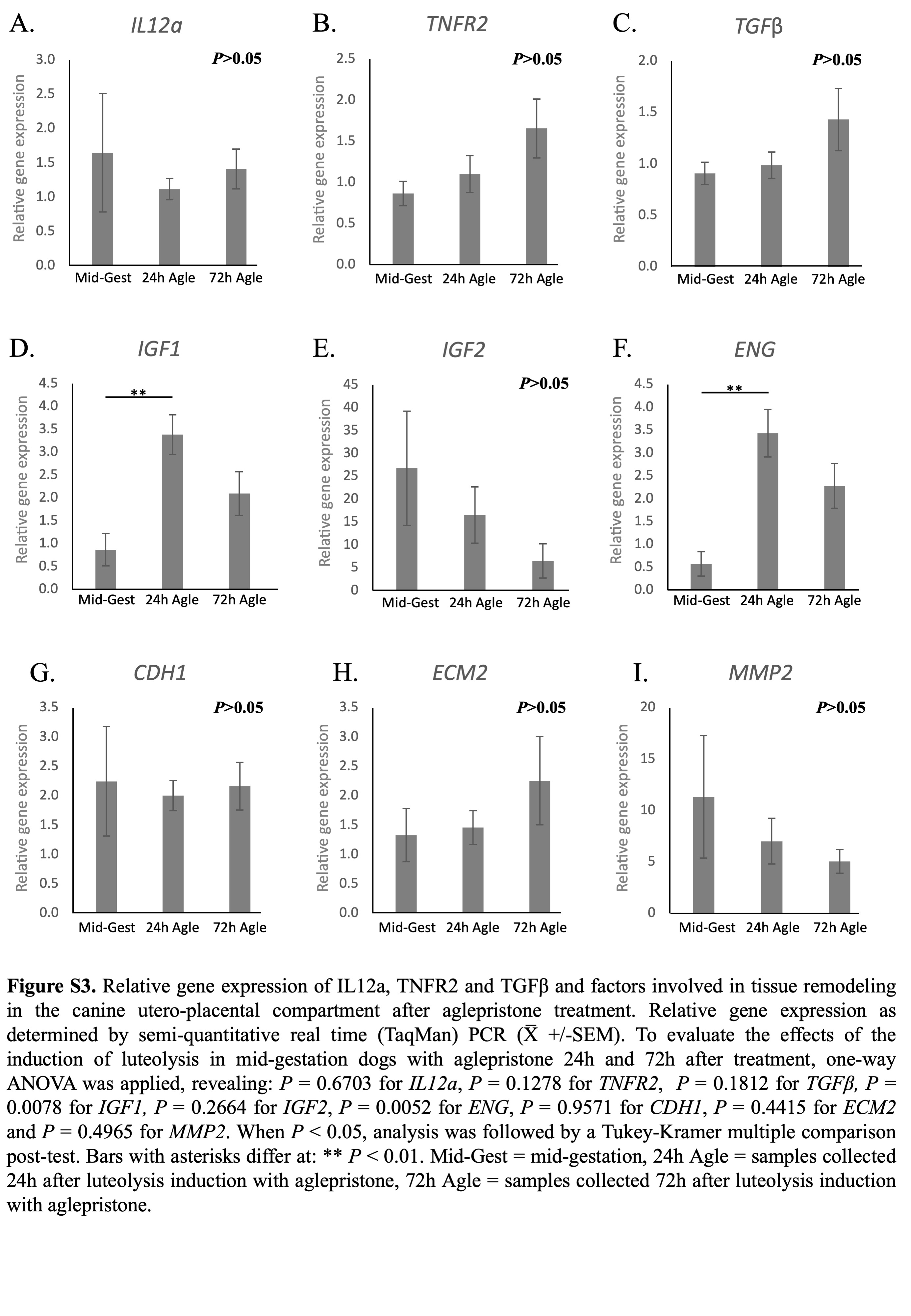

Supplement: Supplementary file 1 [file animals-11-03598-s001.zip › Sup figures/Figure S3.jpeg]
